# Transperineal ultrasound to estimate the appropriate ring pessary size for women with pelvic organ prolapse

**DOI:** 10.1007/s00192-021-04975-9

**Published:** 2021-09-29

**Authors:** Claudia Manzini, Mariëlla I. J. Withagen, Frieda van den Noort, Anique T. M. Grob, Carl H van der Vaart

**Affiliations:** 1 Department of Obstetrics and Gynecology, University Medical Centre Utrecht, Utrecht, The Netherlands; 2 Bergman Clinics, Women’s Health Care, Hilversum, The Netherlands; 3 Robotics and Mechatronics, Faculty of Electrical Engineering Mathematics and Computer Science, Technical Medical Centre, University of Twente, Enschede, The Netherlands; 4 Multi-Modality Medical Imaging, Faculty of Science and Technology, Technical Medical Centre, University of Twente, Enschede, The Netherlands

**Keywords:** Pelvic organ prolapse, Pessary fitting, Ring pessary size, Levator avulsion, Transperineal ultrasound

## Abstract

**Introduction and hypothesis:**

The objective was to predict the successful ring pessary size based on the levator hiatal area (HA).

**Methods:**

This is a prospective case–control study. Women with symptomatic pelvic organ prolapse (POP) choosing pessary treatment were included. All women underwent an interview, clinical examination, and 3D/4D transperineal ultrasound (TPUS). The ring pessary size used in each trial and the reason for unsuccessful trials were recorded. In addition, levator hiatal area divided by ring pessary size (HARP ratio) was measured at rest, maximum contraction, and maximum Valsalva. The HARP ratios of successful and unsuccessful trials were compared, receiver operating characteristic curves in the prediction of successful trials were constructed, and the cut-off optimizing sensitivity and specificity was identified.

**Results:**

A total of 162 women were assessed and 106 were included with 77 successful trials, 49 unsuccessful trials owing to dislodgment or failure to relieve POP symptoms, and 20 unsuccessful trials owing to pain/discomfort. Rest HARP ratio and Valsalva HARP ratio were significantly smaller in the successful trials versus dislodgment/failure to relieve POP symptoms trials (mean rest HARP ratio [SD]: 2.93 [0.59] vs 3.24 [0.67], *p* = 0.021; median Valsalva HARP ratio (IQR): 4.65 (1.56) vs 5.32 (2.08), *p* = 0.004). No significant difference was observed between pain/discomfort trials and successful trials. The best cut-off for the prediction of successful trials was Valsalva HARP ratio ≤ 5.00.

**Conclusions:**

Unsuccessful fitting trials due to dislodgment/failure to relieve POP symptoms are associated with a small ring pessary with respect to the levator HA. A ring pessary that produces a Valsalva HARP ratio > 5.00 has a higher risk of dislodgment/failure to relieve POP symptoms.

## Introduction

Vaginal pessary is a widely used conservative treatment for pelvic organ prolapse (POP) [1, 2]. In clinical practice, the challenge is finding the right pessary that suits an individual woman ideally within the first trial. This process of pessary fitting is based on clinical examination and proceeds by trial and error [3].

Two recent studies have been published on the association between transperineal ultrasound (TPUS) parameters and (un)successful ring pessary fitting [4, 5]. In the study by Cheung et al. [4], successful fitting was compared with unsuccessful fitting owing to pessary dislodgment. A positive significant association was observed between dislodgment and larger levator hiatal area (HA), as well as levator ani muscle (LAM) avulsion. In the study by Turel Fatakia et al. [5], successful fitting was compared with unsuccessful fitting (without distinction between reasons for failure). A positive significant association was observed between unsuccessful fitting and larger levator HA on Valsalva. However, besides the variation in levator HA dimension, variation in ring pessary size should also be considered. An unsuccessful pessary fitting because of dislodgment or failure to relieve POP symptoms may be due to a pessary size that is too small for a given levator HA. On the contrary, an unsuccessful pessary fitting because of pain/discomfort may be due to a pessary size that is too big for a given levator HA. If these assumptions are correct, measuring levator HA could be of added value in estimating the appropriate ring pessary size.

In this study we set out to compare the relative dimension of the ring pessary with respect to the levator HA between successful and unsuccessful pessary fitting trials and to predict the successful ring pessary size based on the levator HA.

## Materials and methods

The data used in the current study were collected as a subset within the GYNecological Imaging using 3D UltraSound (GYNIUS) project on the assessment of pelvic floor contractility with TPUS, which was conducted at our tertiary urogynecological clinic. Women were included in the GYNIUS project between May 2018 and December 2019. The Medical Research Ethics Committee (MREC) exempted the project from ethical approval (reference 18/215), because TPUS was part of our routine diagnostic procedure and standard care. All women signed informed consent forms.

### Inclusion and exclusion criteria

This was a prospective case–control study. Women with symptomatic POP choosing pessary treatment were included. Women who were already using a pessary at intake assessment and those who started the pessary fitting process more than 4 weeks after intake ultrasound assessment were excluded. All women underwent an interview, clinical examination, and 3D/4D TPUS. POP was assessed using the Pelvic Organ Prolapse Quantification system (POPQ) [6].

### Pessary fitting

Pessary fitting was performed according to our standard clinical practice, similar to that described in the literature [7–12], in which the appropriate pessary size is estimated based on clinical examination (i.e., POPQ and digital assessment of fornix posterior width and LAM support). “Fitting trial” was defined as the event of a woman being fitted with a specific ring pessary size, leaving the clinic with the pessary in place, and attending the 2- to 4-week follow-up, in which the success of the fitting trial was assessed. Only fitting trials of ring pessaries (with or without support) were assessed, because the ring pessary is the type most commonly used in our clinic (CooperSurgical®, Milex® pessaries). A fitting trial was considered successful if the specific ring pessary size the woman was fitted with was still in situ at follow-up, if she was satisfied with it, and if she decided to continue using it. On the contrary, if she decided not to continue using the specific ring pessary size she was fitted with, it was considered unsuccessful. In this case, the woman was asked which one of the following was the reason for failure: dislodgment (defined as a pessary that did not stay in place because it fell down or was expelled), failure to relieve POP symptoms, pain/discomfort, increased/de novo urinary incontinence, or other reasons [7]. In the case of unsuccessful fitting, the woman was offered an additional fitting trial with an adjusted ring pessary size. If she agreed, a new pessary was inserted and a 2- to 4-week follow-up was scheduled. One patient could thus have more than one fitting trial, each one with a different ring pessary size. This process continued until an appropriate ring pessary size was found or until pessary treatment was considered not suitable for the woman and a different treatment was chosen. For every fitting trial, the size of the pessary diameter was recorded in centimeters.

### TPUS acquisition and assessment

At intake, TPUS was performed in supine position after bladder emptying. Women were instructed to perform maximal pelvic floor contraction and maximal Valsalva maneuver according to the method described by Dietz [13]. We used a Philips Epiq 7G machine with a X6–1 transducer covered with a gel pad 2 cm thick, and a glove. The gel pad was used to create more distance between the transducer and the woman, so that the LAM could be fully visible within the opening angle on the coronal plane. TPUS volumes analyzed in the current study were acquired without pessary in situ.

An in-house tool was developed in MeVisLab 3.0.2. [14] for TPUS volume assessment, which was done by one observer (CM) blinded to all clinical data. As described in the literature [15], hiatal area at rest (HArest), maximal pelvic floor contraction (HActx), and maximal Valsalva maneuver (HAval) were manually segmented at the plane of minimal hiatal dimensions. In addition, the presence of LAM avulsion was assessed on volumes obtained at maximum contraction. Complete avulsion was defined as a levator–urethra gap of ≥25 mm on the three central slices and could be unilateral or bilateral [15].

### Levator HA to pessary size ratio and statistical analysis

The levator hiatal area to pessary size (HARP) ratio was calculated as levator HA (cm^2^) divided by ring pessary size (cm). The HARP ratio at rest (rest HARP ratio), maximum contraction (contraction HARP ratio), and maximum Valsalva (Valsalva HARP ratio) were calculated for each fitting trial. To the best of our knowledge, no studies have been published in which the HARP ratio is used. Therefore, no formal sample size could be calculated, and this work should be considered an exploratory study.

Rest HARP ratio, contraction HARP ratio, and Valsalva HARP ratio of successful trials and unsuccessful trials, which were separately analyzed based on the reason for failure, were compared. A Welch’s ANOVA and a Games–Howell post hoc test were used if the data were normally distributed and if there were no outliers, but the assumption of homogeneity of variances for a one-way ANOVA was violated. A Kruskal–Wallis test was run if the data were not normally distributed or if there were outliers in the data. Receiver operating characteristic (ROC) curves of the HARP ratios were constructed in the prediction of successful trials. As the dimension of levator HA can be influenced by the presence of complete avulsion [16], ROC curves were also constructed for trials of women with and without complete avulsion. In addition, the cut-off that optimized sensitivity and specificity was identified. Based on this cut-off two groups were defined (i.e., HARP ratio ≤ cut-off and HARP ratio > cut-off) and their association with fitting trial success was tested with a Chi-squared test. Last, the relative risk (RR) of an unsuccessful/successful trial based on the HARP ratio ≤ cut-off or > cut-off was calculated. The statistical analysis was conducted using IBM v 27 SPSS software.

## Results

**Fig. 1:**
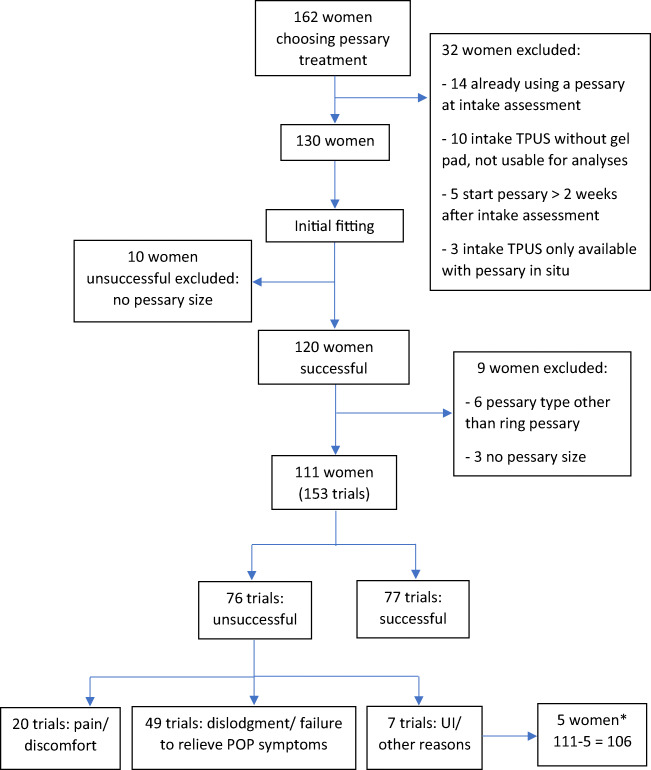
Flow chart with the number of women, number of successful and unsuccessful fitting trials, and reasons for unsuccessful fitting trial. *5 women only underwent unsuccessful trials owing to de novo/increased urinary incontinence or for “other reasons.” As these trials were not analyzed, a total of 106 women (111 – 5) were included in the analysis. *POP* pelvic organ prolapse, *TPUS* transperineal ultrasound, *UI* urinary incontinence

Figure 1 shows the flow chart with the number of women, number of successful and unsuccessful trials, and reasons for unsuccessful trials. Only 5 trials were unsuccessful owing to de novo/increased urinary incontinence and 2 trials for “other reasons.” Because of the small sample size, they could not be separately analyzed and the women who only underwent unsuccessful trials owing to de novo/increased urinary incontinence or for “other reasons” were not included in the analysis. Therefore, 146 trials of 106 women were included in the analysis, with 77 successful trials, 49 unsuccessful trials owing to dislodgment or failure to relieve POP symptoms, and 20 unsuccessful trials owing to pain/discomfort. Of the 106 women included, 49 underwent only one successful trial, 17 only one unsuccessful trial, 28 more than one trial with the last being successful, and 12 more than one trial with the last being unsuccessful.

In Table 1 the demographical, clinical, and TPUS characteristics of the women included are reported.

**Table 1:** Demographical, clinical, and transperineal ultrasound characteristics (*n* = 106)

Parameter	Value
Age, median (IQR)	62.0 (14)
BMI, median (IQR)	24.2 (5.2)
Post-menopausal, *n* (%)	81 (76.4)
Vaginal parity, *n* (%)	104 (98.1)
Assisted vaginal delivery, *n* (%)	9 (8.5)
Prior hysterectomy*, *n* (%)	13 (12.3)
Prior POP surgery (hysterectomy excluded), *n* (%)	9 (8.5)
Predominant compartment POP, *n* (%)	
Anterior	63 (59.4)
Apical	7 (6.6)
Posterior	8 (7.5)
Anterior, apical	3 (2.8)
Anterior, posterior	17 (16.0)
Apical, posterior	3 (2.8)
Anterior, apical, posterior	5 (4.7)
POP stage, *n* (%)	
I	1 (0.9)
II	60 (56.6)
III	45 (42.5)
HA rest (cm^2^), median (IQR)	20.13 (6.61)
HA contraction (cm^2^), median (IQR)	16.93 (5.31)
HA Valsalva (cm^2^), mean (SD)	33.64 (9.85)
Complete avulsion, *n* (%)	42 (39.6)

*BMI* body mass index, *HA* hiatus area, *IQR* interquartile range, *POP* pelvic organ prolapse

^a^4 women (30.8%) underwent a hysterectomy for POP

Ring pessary sizes used ranged from 5.7 cm to 8.9 cm. In Table 2 the comparison of the rest HARP ratio, contraction HARP ratio, and Valsalva HARP ratio between groups is shown. Rest HARP ratio and Valsalva HARP ratio were significantly smaller in the successful trials than in those in which there was dislodgment/failure to relieve POP symptoms (*p* = 0.021 and 0.004 respectively). Contraction HARP ratio was not significantly different between groups. Therefore, the post hoc test was not performed. No statistically significant difference was observed between pain/discomfort trials and successful trials.

**Table 2:** Comparison of rest HARP ratio, contraction HARP ratio, and Valsalva HARP ratio between groups. A Kruskal–Wallis test was run if not otherwise specified

Parameters	Group 1: successful (*n* = 77)	Group 2, dislodgment/failureto relieve POP symptoms (*n* = 49)	Group 3: pain/discomfort(*n* = 20)	Comparison	Significance
Rest HARP ratio, mean (SD)	2.93 (0.59)	3.24 (0.67)	3.20 (0.99)	All groups	0.027*
				Group 1 vs 2	0.021**
				Group 1 vs 3	0.483**
				Group 2 vs 3	0.979**
Contraction HARP ratio, median (IQR)	2.42 (0.67)	2.59 (0.72)	2.44 (1.01)	All groups	0.116
Valsalva HARP ratio, median (IQR)	4.65 (1.56)	5.32 (2.08)	4.60 (2.46)	All groups	**0.006**
				Group 1 vs 2	0.004***
				Group 1 vs 3	1.000***
				Group 2 vs 3	0.605***

Bold indicates the stastistically significant parameters

*Welch’s ANOVA

**Games–Howell post hoc test

***Bonferroni correction for multiple comparison

Receiver operating characteristic curves of the HArest and the HAval were constructed in the prediction of successful trials versus those in which there was dislodgment/failure to relieve POP symptoms. In addition, sub-analyses were made for the trials of women without and with complete avulsion (Table 3). When no distinction was made based on the presence of complete avulsion, the AUC of HAval was 0.67 (0.58–0.77) and the best cut-off in the prediction of successful fitting was 5.00. In the case of complete avulsion, the AUC of HAval was 0.79 (0.65–0.92) and the best cut-off was 5.13. Applying the cut-off of 5.00 of the whole group to the group of women with complete avulsion, sensitivity and specificity were 0.67 and 0.84 respectively.

**Table 3:** Area under the receiver operating characteristic curve (AUC) of the rest HARP ratio and the Valsalva HARP ratio in the prediction of successful trials versus those in which there was dislodgment/failure to relieve POP symptoms. HARP ratio = levator hiatal area to ring pessary size

Trials	Parameter (cm)	AUC (95% CI)	*p* value	Best cut-off^a^
All successful trials or those in which there was dislodgment/failure to relieve POP symptoms (*n* = 126)	Rest HARP ratio	0.63 (0.53–0.73)	**0.017**	HAval/pessary size ≤ 5.00 (sensitivity 0.68, specificity 0.67)
	Valsalva HARP ratio	0.67 (0.58–0.77)	**0.001**	
Trials of women without complete avulsion (*n* = 77)	Rest HARP ratio	0.65 (0.51–0.78)	**0.039**	HArest/pessary size ≤ 2.94 (sensitivity 0.59, specificity 0.63)
	Valsalva HARP ratio	0.59 (0.45–0.73)	0.222	
Trials of women with complete avulsion (*n* = 49)	Rest HARP ratio	0.56 (0.39–0.72)	0.497	HAval/pessary size ≤ 5.13 (sensitivity 0.79, specificity 0.72)
	Valsalva HARP ratio	0.79 (0.65–0.92)	**0.001**	

Bold indicates the stastistically significant parameters

Sensitivity (i.e., of all successful trials, percentage that the model predicts as successful)

Specificity (i.e., of all trials in which there was dislodgment/failure to relieve POP symptoms, percentage that the model predicts as trials in which there was dislodgment/failure to relieve POP symptoms)

*HAval* maximal Valsalva maneuver, *HArest* hiatal area at rest

^a^Best cut-off in the prediction of successful trials

A Chi-squared test between Valsalva HARP ratio (≤ 5.00 vs > 5.00) and fitting trial (successful vs unsuccessful owing to dislodgment/failure to relieve POP symptoms) showed a statistically significant association between Valsalva HARP ratio ≤ 5.00 and successful trial and Valsalva HARP ratio > 5.00 and unsuccessful trials (*p* = 0.00). 76.5% of the trials with a Valsalva HARP ratio ≤ 5.00 were successful, whereas only 43.1% of the trials with a Valsalva HARP ratio > 5.00 were successful. Trials with Valsalva HARP ratio > 5.00 had a RR of 2.42 (1.49–3.92) of being unsuccessful owing to dislodgment/failure to relieve POP symptoms. The RR was 3.62 (95% CI 1.47–8.95) in the case of trials of women with complete avulsion.

Of the 28 women who underwent one or more trials before being successful, 23 had a first unsuccessful trial owing to dislodgment/failure to relieve POP symptoms. In this group a Chi-squared test between Valsalva HARP ratio (≤ 5.00 vs > 5.00) and fitting trial (first unsuccessful versus last successful) showed a statistically significant association between Valsalva HARP ratio > 5.00 and first unsuccessful trials and Valsalva HARP ratio ≤ 5.00 and last successful trial (*p* = 0.02). In the first unsuccessful trial 56.5% of the women had a Valsalva HARP ratio > 5.00 and 43.5% a Valsalva HARP ratio ≤ 5.00. In the last successful trials 21.7% of the women had a Valsalva HARP ratio > 5.00 and 78.3% a Valsalva HARP ratio ≤ 5.00. Trials with Valsalva HARP ratio ≤ 5.00 had a RR of 2.31 (1.05–5.12) of being successful.

Furthermore, 17 women underwent only unsuccessful trials owing to dislodgment/failure to relieve POP symptoms (10 women one trial, 6 women two trials, and 1 woman three trials). Of these, 14 women (82.4%) received exclusively pessaries that were too small according to our cut-off.

## Discussion

In the case of unsuccessful trials owing to dislodgment/failure to relieve POP symptoms, ring pessaries are too small with respect to the levator HA. A ring pessary size that produces a Valsalva HARP ratio > 5.00 has a higher risk of dislodgment and failure to relieve POP symptoms.

As hypothesized, the HARP ratio was significantly bigger in the case of trials in which there was dislodgment/failure to relieve POP symptoms than in successful trials. As levator HA is determined by the status of the LAM, these results suggest that LAM support might play an important role in holding ring pessaries in place. Currently, pessary fitting is based on POPQ and digital assessment of fornix posterior width and LAM support [7–12]. In this process, the dimension of the levator hiatus is not formally measured. This could (partially) explain the relatively high rate of unsuccessful pessary fitting, reported to be as high as 59% [12].

When no distinction was made between complete avulsion and no avulsion, the AUC of the Valsalva HARP ratio was 0.67. In the case of complete avulsion, the Valsalva HARP ratio showed an almost excellent level of discrimination (according to Hosmer et al. [17]). 76.5% of the trials with a Valsalva HARP ratio ≤ 5.00 were successful, whereas only 43.1% of the trials with a Valsalva HARP ratio > 5.00 were successful, with an RR of 2.4 of being unsuccessful (RR of 3.62 in the case of complete avulsion). By analyzing women who underwent a first unsuccessful trial owing to dislodgment/failure to relieve POP symptoms and a last successful trial, we observed an RR of 2.31 of being successful in the case of a Valsalva HARP ratio ≤ 5.00, compared with a ratio > 5.00. These results suggest that measuring the Valsalva HARP ratio could allow for a faster selection of the successful size, thus reducing the need for extra visits for pessary refitting and the discomfort due to multiple fitting trials. After the disappointment of one or more unsuccessful trials, some women refuse to undergo an additional one, thus missing the chance of a successful fitting. In these cases, a faster selection of the successful size could increase the pessary fitting success rate by reducing the number of unsuccessful trials.

In our study 82.4% of the women who only underwent unsuccessful trials owing to dislodgment/failure to relieve POP symptoms received exclusively pessaries that were too small: if our cut-off were applied, the pessary fitting success rate could have been higher or the women could have been spared unnecessary trials. A comparative study, with a sample size based on our data, is needed to confirm that using the Valsalva HARP ratio for selecting the pessary size does indeed reduce the need for pessary refitting and increases the chance of a successful initial fitting.

No significant difference was observed between successful trials and unsuccessful trials owing to pain/discomfort. This suggests that pain and discomfort might not be related to the size of the pessary with respect to the levator HA. However, complications such as pain/discomfort and vaginal bleeding due to ulceration might be related to the size of the pessary with respect to the vaginal space. Future studies should test this hypothesis and focus on a quantitative method to assess the maximal pessary size that can be placed without causing the complications mentioned above. Knowing the minimal ring pessary size that is likely to stay in place (through the HARP ratio) and the maximal ring pessary size that is unlikely to cause complications, would help the clinician to estimate whether a ring pessary is a good option for a specific woman or not.

Literature on the association between anatomical parameters and pessary size is very limited: Nager et al. showed that POPQ measures do not predict the incontinence pessary size in women with POP stage ≤2 [18].

Strengths of our study include the prospective design, which reduced the risk of selection bias. All scans and TPUS assessments were performed by the same clinician, thus reducing a source of variability. In addition, TPUS assessment was performed blinded to all clinical data and to the pessary size. Some limitations have to be acknowledged. HARP ratio analyses are only applicable to fitting trials performed with one pessary type because different pessary types cannot be compared. We selected the ring pessary because it is the pessary type most commonly used in our clinic. An additional limitation is that the generalizability of the results might be limited because the study was conducted in a urogynecological center where primary care is not provided.

In conclusion, unsuccessful fitting trials owing to dislodgment or failure to relieve POP symptoms are associated with a small ring pessary with respect to the levator HA: a ring pessary size that produces a Valsalva HARP ratio > 5.00 has a higher risk of dislodgment and failure to relieve POP symptoms. These results suggest that TPUS might be of added value in the ring pessary-fitting process.
